# Attitudes of anesthesiologists towards implementation of PENG block in non-operative treatment of hip fractures in the Netherlands: a national survey study

**DOI:** 10.1186/s12871-026-03957-y

**Published:** 2026-05-29

**Authors:** Marte I. Lommerse, Daphne van Embden, Thamar Kroes, Rachel J.H. Smits, Jan Willem Kallewaard, Hanna C. Willems, Henk J. Schuijt, the Dutch PENG Study Group, Bart Spaetgens, Detlef Van der Velde, Esther M. M. Van Lieshout, Gezina T. M. L. Oei, Glenn Van de Vossenberg, Han Hegeman, Hugo H. Wijnen, Job N. Doornberg, Johan Haumann, Koen Bos, Lennart G. Wasmoeth, Leon Timmerman, Michiel H. J. Verhofstad, Miriam Van der Velden, Nicole Lefel, Pieter Joosse, Renée A. G. Brüggemann, Rutger Zuurmond, Seppe J. S. H. A. Koopman, Selina E. I. Van der Wal, Taco Gosens

**Affiliations:** 1 Department of Trauma Surgery, Amsterdam University Medical Center – location AMC, Meibergdreef 9, 1105 AZ Amsterdam, the Netherlands; 2 Geriatrics Section, Department of Internal Medicine, Amsterdam University Medical Center – location AMC, Meibergdreef 9, 1105 AZ Amsterdam, the Netherlands; 3 Amsterdam Bone Center/Amsterdam Musculoskeletal Sciences, Amsterdam University Medical Center Research Institute, Meibergdreef 9, 1105 AZ Amsterdam, the Netherlands; 4 Department of Trauma Surgery, St. Antonius Ziekenhuis, Soestwetering 1, 3543 AZ Utrecht, the Netherlands; 5 Department of Anesthesiology, Pain- and Palliative Medicine, Radboud University Medical Center, Geert Grooteplein Zuid 10, 6525 GA Nijmegen, The Netherlands; 6 Department of Anesthesiology and Pain Medicine, Rijnstate Ziekenhuis, Wagnerlaan 55, 6815 AD Arnhem, The Netherlands; 7 Department of Anesthesiology and Pain Medicine, Amsterdam University Medical Center – location AMC, Meibergdreef 9, 1105 AZ Amsterdam, the Netherlands

**Keywords:** PENG block, Phenol neurolysis, Hip fracture, Palliative care, Implementation science

## Abstract

**Background:**

Effective pain management in frail patients with hip fractures who are not candidates for surgery remains challenging. The pericapsular nerve group (PENG) block with phenol has been proposed as a minimally invasive option in this context. However, its integration into palliative care pathways is not well established. This study evaluated how Dutch anesthesiologists perceive the PENG block with phenol for non-operative pain management and explored the current state of its implementation.

**Methods:**

We conducted a national cross-sectional survey among all registered anesthesiologist-pain specialists in the Netherlands (N = 192). The survey assessed perceived barriers and facilitators to implementation, categorized at the system, staff, and intervention levels. Secondary outcomes included perceptions of acceptability, feasibility, and appropriateness.

**Results:**

Sixty anesthesiologists completed the survey (adjusted response rate 31%), of whom 88% reported prior experience with the PENG block using phenol. Reported barriers included uncertainty about long-term efficacy (57%), logistical constraints (48%), and limited training opportunities (35%). Facilitators were the availability of clear protocols (55%) and an expanding evidence base (50%). Respondents largely considered the block technically straightforward and clinically appropriate, particularly for femoral neck fractures. Nevertheless, feasibility concerns were highlighted regarding referral pathways and interdepartmental collaboration.

**Conclusions:**

Dutch anesthesiologists widely accept the PENG block with phenol as an appropriate option for pain management in selected frail patients with hip fractures in the non-operative setting. Implementation is currently constrained by evidence gaps, logistical barriers, and institutional variability. Targeted staff-, intervention-, and system-level strategies may facilitate broader adoption, including structured residency training, institutional protocols with patient education tools, and interdisciplinary collaboration across relevant specialties. Further evidence and national guideline development are needed to facilitate standardized practice and international implementation.

**Supplementary Information:**

The online version contains supplementary material available at 10.1186/s12871-026-03957-y.

## Background

The pericapsular nerve group (PENG) block is a novel regional anesthesia technique that targets the articular sensory branches of the femoral and obturator nerves [1]. It is performed under ultrasound guidance, delivering local anesthetic between the anterior inferior iliac spine and iliopubic eminence. As it provides effective analgesia without significant motor blockade [2], the PENG block may be a promising development for both perioperative and nonoperative pain management in patients with hip pathology. Recent literature describes the PENG block in various settings for hip analgesia. Examples include early analgesia of hip fractures, postoperative analgesia after hip arthroplasty and arthroscopy, as well as pelvic fracture surgery and pediatric open hip surgery [3–7]. Nonoperative applications include chronic hip pain and hip fractures. In the latter, PENG has been combined with alcohol or phenol for neurolysis, which results in long lasting pain relief of approximately 6–12 weeks [8–11].

In the Netherlands, the recent development of neurolysis in nonoperatively managed patients after hip fractures reflects a shift in clinical discourse surrounding end-of-life care of the frailest older patients. Hip fractures remain a growing concern in frail older adults, due to advanced age and multiple comorbidities [12, 13]. Recent literature suggests that surgical intervention may not always be the best option and nonoperative management could be considered in an end of life care setting [14]. In these cases, the focus shifts to the quality of dying, aiming for comfort and effective pain management [15]. For this purpose, chemical denervation through a PENG block is considered a promising intervention for anesthesiologists and pain specialists, providing better analgesia with a reduction of opioid use [16].

The use of the PENG block with phenol in the palliative setting in the Netherlands is still in “early-stage” implementation, progressing gradually from regional introduction to nationwide integration in care pathways [10]. To further current clinical implementation efforts and facilitate broader adoption of this technique, a thorough understanding of the implementation is required. This study evaluates implementation determinants and implementation outcomes, specifically acceptability, feasibility and appropriateness [17]. The following research question will be answered: ‘What barriers and facilitators do anesthesiologists-pain specialists identify in regard to implementation of PENG block with phenol for pain management in non-operative patients with hip fractures?’ Insights into these determinants will be used to evaluate and recalibrate implementation efforts.

## Methods

This study employs a cross-sectional survey study design with quantitative and qualitative items to provide a comprehensive understanding of anesthesiologists' attitudes, experiences and perceptions regarding the use of PENG block with phenol in palliative care settings. The study protocol is attached in *Supplementary file 1*. This study was conducted in accordance with the ethical principles outlined in the Declaration of Helsinki and was deemed not subject to the Medical Research Involving Human Subjects Act by the Medical Ethics Review Committee (METC) of the Amsterdam University Medical Center, the Netherlands (reference number 2025.0116). Informed consent was obtained electronically; participants were presented with detailed study information and were required to actively tick a consent box before proceeding to any survey questions. For quality reporting, the Checklist for Reporting of Survey Studies (CROSS) guidelines as well as the Standards for Reporting Implementation Studies (StaRI) guidelines were followed [18, 19].

### Survey population

In this study, eligible participants were anesthesiologists-pain specialists as registered in the Dutch national medical specialist registry (BIG-register), in order to reduce coverage error. All registered specialists were considered potential survey participants, regardless of their experience with the PENG block in order to reduce and sampling error [20]. Partial surveys were included in the analysis if baseline demographic questions had been answered in order to maximize data utilization while preserving the interpretability of the study population characteristics.

### Theoretical frameworks for study and survey design

To evaluate implementation of PENG block in a hospital setting, this study applied two complementary frameworks, guided by the taxonomy of theoretical approaches in implementation science [21]. First, a determinant framework by Geerligs et al. (2018) was used to identify barriers and facilitators in three domains: system, staff or intervention [22]. The underlying theory of this framework posits that implementation is influenced by determinants at the level of system, staff, and intervention. Barriers and facilitators must be understood in relation to these levels. This multi-level perspective informs the design of both the study and the survey, as well as guiding the synthesis presented in this article. Second, the evaluation framework by Proctor et al. [17] was used to evaluate PENG block implementation outcomes. In this context, determinants refer to the factors that influence implementation, while implementation outcomes reflect how well the implementation is proceeding. The three implementation outcomes selected were:

- Acceptability: How well PENG block is received by anesthesiologists.
- Feasibility: The practicality of implementing PENG block within the hospital setting.
- Appropriateness: The perceived relevance and suitability of PENG block for managing pain in non-operative managed hip fracture patients.

The rationale for selecting these outcomes was that they are generally considered “early-stage” implementation outcomes, meaning that they are measured before widespread adoption of new interventions. Implementation costs could be considered the fourth “early-stage” implementation outcome in the evaluation framework by Proctor et al., but this was beyond the scope of this study [17].

### Survey development

With these theoretical implementation frameworks as its foundation, survey questions were developed and structured in terms of determinants (e.g. barriers and facilitators) or implementation outcomes (e.g. acceptability, feasibility or appropriateness). In order to minimize the risk of measurement error, and to increase survey validity and reliability [20, 23], the survey questions were developed by 5 authors (TK, RS, ML, HJS & HW) and piloted by 2 anesthesiologist-pain specialists (RS and JWK). The survey started with demographic questions (age, specialism (i.e. anesthesiologist-pain specialist or anesthesiologist with another subspecialty), years of experience and previous PENG block experience). After these baseline questions, the survey questions were mainly quantitative of nature (e.g. Likert-scale questions, close-ended questions, and multiple-choice questions). Survey questions were coded using the AIM-IAM-FIM psychometric assessment for the three corresponding implementation outcomes [24]. An overview of the implementation outcomes and how they have been addressed in the survey questions can be found in *Supplementary file 2*. The questionnaire was then divided according to experience with the PENG block, resulting in two final surveys that consisted of 26 and 16 questions in total for anesthesiologist-pain specialists with and without experience, respectively. The two surveys as well as the coding sheet for each question according to implementation outcome can be found in *Supplementary file 3a-b*.

### Data sampling

Participant recruitment conform voluntary response and snowball sampling was conducted through the use of an online survey and with the support of a national PENG-consortium. A weblink redirecting to the online survey was distributed via the Dutch National Anesthesiology Society (Nederlandse Vereniging voor Anesthesiologie, NvA), through a LinkedIn group dedicated to anesthesiology research, as well as via e-mail to maximize coverage. Two and four weeks after initial distribution, reminders were sent to encourage participation and reduce nonresponse bias. Responses were collected from the 25th of February until the 28th of April 2025. For survey distribution, data collection and data management Qualtrics XM software, Version March 2025 (Qualtrics, Provo, UT) was used. Multiple submissions from the same participant were prevented by Qualtrics through automatic blocking of repeat entries from the same browser or device after survey completion. All responses were collected anonymously, with no identifying information stored or linked to participants. The Qualtrics XM survey system was only available to one researcher (ML) and was secured with two-step authentication to ensure data confidentiality.

### Statistical analysis

Quantitative survey data were analyzed using descriptive statistics. To summarize continuous respondent characteristics and survey item variables, descriptive methods were used. Normal distribution was assessed based on histogram plotting. In the case of a skewed distribution, median and interquartile ranges (IQRs) were used. Multiple choice questions were reported with counts and percentages. Responses to Likert-scale questions were treated as ordinal data and summarized using medians and IQRs. No modification of variables was performed prior to analysis. No weighting or propensity score adjustments were applied due to the small sample size. Missing data were not imputed; analyses were performed on available cases only. Non-response error was not evaluated, as response rate estimation was not possible due to the method of survey distribution and anonymity of recipients. All analyses were performed using SPSS software version 28 (IBM Corporation).

## Results

### Survey response rate

The survey was distributed to all 192 registered anesthesiologist-pain specialists in the Netherlands. As the survey was distributed via open anonymous weblinks, the number of unique visitors and corresponding view or participation proportions could not be determined. A total of 60 responses were received, of which 58 were fully completed resulting in a completion proportion of 97%. Using the American Association for Public Opinion Research (AAPOR) standard definitions, the adjusted response rate was 30% (RR1, completed surveys only) and 31% (RR3, including partial surveys) [25].

### Demographic details of survey participants

From the 60 eligible respondents, 53 had previous experience with PENG with phenol in non-operatively treated patients after hip fractures and 7 respondents had no experience with this technique (88% versus 12%). Respondents were predominantly anesthesiologist-pain specialists (67%), with a median of 11 years of clinical experience (IQR 6–25). Respondents worked in hospitals located in the middle and southern regions of the Netherlands, with no survey participation from hospitals in the most northern provinces. All baseline characteristics of the study participants have been summarized in Table 1*.*

**Table 1:** Demographics and clinical experience of all survey participant

Characteristic	**All respondents** **(N = 60)**	**With PENG block experience (N = 53)**	**Without PENG block experience (N = 7)**	**Missing (%)**
Median age in years (IQR)	43.0 (37–53)	39.5 (37–52.3)	58.0 (49–60)	1 (1.7)
Pain specialist (%) Other or no subspecialty (%)	40 (67) 20 (33)	33 (62) 20 (38)	7 (100) 0 (0)	0 0
Median years of clinical experience (IQR)	11 (6–25)	10.0 (5–17.5)	25.0 (15–34)	0

### Personal experience with the PENG block

Among respondents with experience in performing the PENG block, 93% had performed the procedure at least 5 times. Respondents were primarily experienced in performing the block on patients with femoral neck fractures. All participants primarily used the single injection technique, and 79% used 7–10 ml phenol. Nearly half of the respondents reported feeling competent after 1–3 procedures, while a similar proportion gained confidence after 4–6 procedures (42% each). Most often reported training sources included colleagues (83%), scientific publications (76%), and instruction or tutorial videos (57%). Table 2 outlines key characteristics of PENG block with phenol as currently implemented by respondents.

**Table 2:** Overview of PENG block application as reported by respondents treating non-operatively managed hip fractures

Characteristic (%)	**With PENG block experience (N = 53)**	**Missing** (%)
No. blocks performed		0
< 5	4 (7.5)	
5–10	16 (30)	
10–20	17 (32)	
> 20	16 (30)	
Most used technique		0
Single injection	53 (100)	
Double injection	0	
Other	0	
Amount of phenol used		0
< 7 ml	1 (1.9)	
7–10 ml	41 (77)	
11–15 ml	8 (15)	
Other	3 (5.7)	
Location of block-placement		2 (3.8)
Operating room	2 (3.8)	
Out-patient clinic room	10 (19)	
Recovery ward	37 (70)	
Other	2 (3.8)	
Indication to block time		2 (3.8)
< 12 h	14 (26)	
12–24 h	28 (53)	
24–36 h	9 (17)	
No. blocks before self-reported competence		2 (3.8)
1–3 blocks	22 (42)	
4–6 blocks	22 (42)	
7–10 blocks	6 (11)	
> 10 blocks	1 (1.9)	
Training sources		2 (3.8)
Colleagues	44 (83)	
Scientific publication	40 (76)	
Scientific presentation	22 (42)	
Instructional video	30 (57)	
Educational course	11 (21)	

### Barriers and facilitators to implementation

**Fig. 1:**
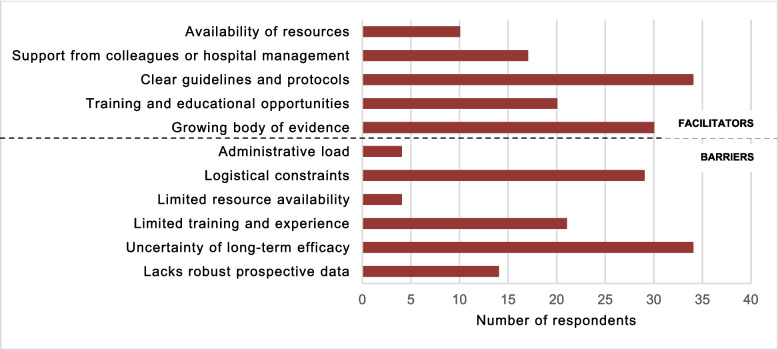
Barriers and facilitators as reported by all survey participants

The most frequently reported barriers included uncertainty of long-term efficacy (57%), logistical constraints (48%) and limited training opportunities (35%). Examples of logistical barriers noted by respondents included the lack of standardized care pathways and the absence of a procedure code in hospital systems for registering the PENG block. Additionally, some participants highlighted challenges in coordinating care across specialties and logistical difficulties in organizing the block for patients who were rapidly discharged or required ambulance transport back to a nursing home. The availability of clear guidelines and protocols (55%) as well as a growing body of scientific evidence (50%) were most frequently reported as important facilitators. A graphic presentation of all barriers and facilitating factors can be found in Fig. 1*.*

### Acceptability

**Fig. 2:**
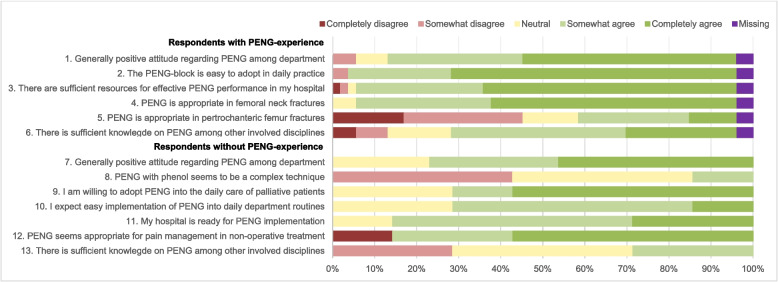
Responses to Likert-scale statements reported by survey participants with and without PENG-experience

To assess acceptability, respondents were asked to rate their agreement with statements regarding the general attitude of their department, ease of application and the complexity of PENG block with phenol (Fig. 2). The general attitude towards the PENG block of departments was most frequently perceived as positive (statement 1 & 7). All respondents agreed that all anesthesiologists should be permitted to perform the PENG block, not exclusively anesthesiologist-pain specialists. Furthermore, 9 participants provided other examples of who could perform the PENG block such as emergency care-doctors, orthopedic-/trauma surgeons, radiologists or nursing home doctors. Among anesthesiologists who already had previous experience with the technique, the majority agreed that the PENG block was easy to adopt in their daily clinical practice (statement 2). In respondents without previous PENG block experience, willingness to adopt the PENG block in palliative care was high (statement 9).

### Feasibility

Feasibility was assessed through survey items addressing availability of resources, time to block placement, ease of integration into daily practice and institutional readiness (Table 2*, *Fig. 2). A majority of respondents with PENG experience indicated that their departments were equipped to implement the technique (statement 3). 53% of respondents reported PENG block placement within 12–24 h after the indication was set, while 26% reported PENG block placement within the first 12 h. Among participants without PENG block experience, expectations regarding ease of implementation were rated neutral or somewhat positive in 86% (statement 10). They also expressed a fairly positive attitude towards the state of institutional readiness for implementation in their hospital (statement 11).

### Appropriateness

Appropriateness was explored via Likert-scale statements addressing whether PENG block with phenol is a suitable technique for managing pain in patients with non-operatively managed hip fractures (Fig. 2). Of respondents with PENG block experience, a higher proportion endorsed appropriateness in femoral neck fractures compared to trochanteric fractures (statements 4 & 5). In the group without PENG block experience, most respondents (86%) rated the technique as somewhat or completely appropriate for hip fracture patients in the palliative setting (statement 12).

## Discussion

**Fig. 3:**
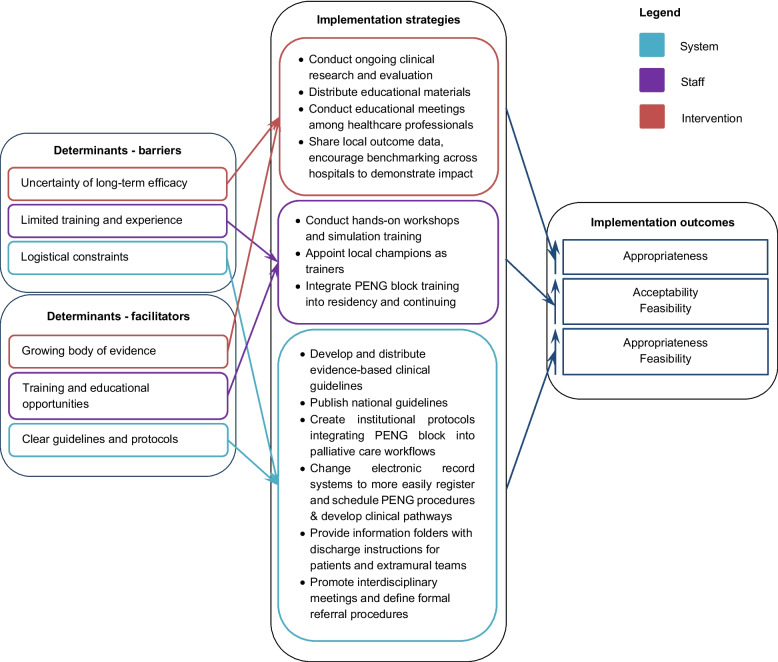
Implementation determinants, strategies, and outcomes for PENG block with phenol in palliative hip fracture care

This national survey explored perspectives of Dutch anesthesiologist-pain specialists on implementing the PENG block with phenol for non-operatively managed hip fractures in palliative care. Most respondents found the block acceptable and appropriate, particularly for femoral neck fractures. However, barriers such as uncertainty about long-term efficacy, limited training and logistical challenges remain. Still, the reported willingness and institutional readiness suggest strong potential for implementation. An overview of matched implementation strategies across system, staff, and intervention domains is presented in Fig. 3.

The large proportion of respondents with PENG block experience (88%) reflects broader adoption of the technique in the Netherlands. This is in line with previous Dutch retrospective research illustrating that PENG block already has become standard of care for palliative care pain management in several Dutch hospitals [10]. However, self-selection of survey participants could also play a role, as reflected by Rogers’ theory of innovation diffusion, where early adopters initiate uptake before broader clinical use [26]. The clusters of survey participants concentrated in middle and south of the Netherlands could reflect the presence of early adopters, promoting the technique within their departments and regional networks.

### Intervention level considerations

The reported barriers and facilitators align with early-stage PENG block implementation in the Netherlands. Uncertainty about long-term efficacy of phenol neurolysis and a lack of robust clinical evidence emerged as evident barriers within the intervention domain [22]. Differing views on the appropriateness of the PENG block for pertrochanteric versus femoral neck fractures further reflect this evidence gap. Though administered in an extracapsular plane, PENG block anesthetizes sensory branches to the joint capsule that innervate intracapsular structures [27]. Analgesia for extracapsular structures like the trochanter likely results from high-volume spread [28]. This suggests that its efficacy for pertrochanteric femur fractures may be less consistent compared to femoral neck fractures. Emphasis om these evidence gaps is expected, given the recent introduction of PENG and the limited number of publications in the setting of nonoperative hip fracture management [29–31]. Survey responses underscore this attention to available evidence to inform physicians and patients. Other implementation strategies at the intervention level should moreover include distributing educational materials, conducting educational meetings among healthcare professionals, sharing local outcome data, and benchmarking across hospitals to demonstrate clinical impact (Fig. 3).

### Staff-level considerations

Respondents found the block acceptable due to its clear anatomical landmarks and predictable effect. The importance of peer-led, hands-on training was repeatedly emphasized, especially for phenol injection and spread nuances. While some learned through publications or videos, most stressed the need for practical exposure. Staff-level strategies include integrating PENG block training into residency and continuing medical education programs, appointing local champions as trainers, and embedding on-site coaching, skills workshops, and mentorship within existing pain or palliative care networks [32]. These approaches aim to enhance procedural skills, confidence, and low-threshold adoption across anesthesiology teams. Embedding the PENG block as a core skill within general anesthesiology practice may reduce reliance on individual providers and improve consistent access across settings.

### System-level considerations

Respondents highlighted the need for national guidelines, institutional protocols, and clear care pathways to standardize procedures and clarify patient selection. Strategies to support system-level adoption therefore include updating electronic record systems to facilitate procedure registration and scheduling, publishing national guidelines, and developing clinical pathways that integrate the PENG block into palliative care workflows. Furthermore, as more analgesic techniques are proposed in patients with hip fractures, the development of a nonoperative pain management decision-making tool may further support clinicians in choosing the most suitable analgesic strategy according to individual patient and fracture characteristics. This need for structured guidance of PENG block adoption is echoed in international literature, which has also raised the question of what clinical knowledge is still required before the PENG block can be broadly recommended in routine practice [33]. Patient and caregiver information folders were proposed as a practical strategy to support implementation and care continuity, particularly in a patient population with limited follow-up opportunities and the emotional burden that could limit patients’ ability to retain verbal information [34]. Furthermore, considering the anesthesiologist’s unique transitional role in the care pathway, caregiver materials may help bridge communication with primary care and home care teams. The successful implementation of the PENG block with phenol will likely also require closer interdisciplinary collaboration between anesthesiology, geriatrics, orthopedics, and emergency medicine [35]. While anesthesiologists play a key role pain management decision-making, several respondents emphasized the need for shared indications and clear referral pathways with referring departments. Some respondents supported expanding the skillset to other specialties in outpatient or nursing home settings to improve feasibility by reducing delays and transfers. However, training and knowledge on the use and side effects of phenol is crucial for patient safety and effectiveness. These suggestions align with the survey’s feasibility findings, which identified logistical constraints and the current state of knowledge among other involved disciplines as bottlenecks. Ensuring 24/7 availability of the PENG block with phenol is an important consideration for successful integration into routine palliative care. Strategies that enable interdisciplinary task-sharing and clarify referral pathways are critical system-level interventions, aimed at improving communication, role clarity, and timely care delivery [36, 37].

### Strengths and limitations

This study has several important strengths. It is the first to systematically explore the implementation process of the PENG block with phenol from the perspective of anesthesiologists, providing novel insights into clinical practice. A key strength lies in the structured linkage of survey findings to concrete implementation strategies, offering actionable guidance for clinical translation. Collectively, these results establish an important foundation for improving palliative care for non-operatively managed hip fracture patients and may serve as a catalyst for future international collaboration aimed at optimizing implementation pathways. However, this study is subject to some limitations. The ARR of 31%, combined with the overrepresentation of respondents with prior PENG block experience and geographic clustering of participants, introduces potential selection- and nonresponse bias and limits generalizability of the results [20]. Furthermore, the overrepresentation of PENG block users may have also led to an overestimation of perceived readiness for implementation, as clinicians in less experienced settings may face additional barriers such as credentialing processes or pharmacy regulations regarding phenol. Moreover, due to the use of open, anonymous survey links, the true number of eligible recipients could not be ascertained, limiting the accuracy of the reported response rate and precluding formal nonresponse analysis. The distribution channels used could potentially lead to underrepresentation of less engaged clinicians. This limitation was considered during the study design phase but was accepted as a pragmatic trade-off to ensure feasibility. However, the potential impact of partial response bias is likely minimal, as reflected by the negligible difference between crude and adjusted response rates (30% versus 31%). As this survey was conducted within the context of a real-world, ongoing implementation project, lower response rates are not unexpected. The relatively small size of the non-experienced group restricted statistical comparisons and further limits generalizability. Finally, data are self-reported and perception-based. Data may therefore be subject to recall and desirability bias. While such perceptual outcomes limit clinical inference of the current study, they are appropriate for this implementation stage and inform further research and implementation strategies. More distal and patient-level outcomes have been evaluated in complementary studies [10, 38].

## Conclusion

Anesthesiologist-pain specialists view the PENG block with phenol as acceptable, feasible and appropriate for pain management in non-operatively managed hip fracture patients in the palliative setting. However, broader adoption in the Netherlands is still in an early phase. The absence of robust prospective data and standardized guidelines are barriers to consistent, evidence-based practice. Despite these barriers, the willingness to adopt the technique and the high procedural confidence among clinicians suggest that implementation is already occurring in a clinician-driven manner. To support safe and sustainable integration, implementation strategies should now prioritize international guideline development and creation of institutional protocols embedding the PENG block into palliative care workflows. Implementation strategies should further emphasize interdisciplinary collaboration across all involved disciplines, alongside structured, hands-on peer training. Sharing local outcome data and benchmarking across hospitals may help demonstrate impact and strengthen the evidence base. Given the relatively rapid uptake of the PENG block with phenol in end-of-life hip fracture care in the Netherlands, national experiences offer valuable insights for international implementation efforts. Our findings underscore the technique’s clinical potential, while also highlighting the importance of embedding its use within a multidisciplinary standardized, and evidence-informed care pathway.

## Supplementary Information

Supplementary Material 1.

Supplementary Material 2.

Supplementary Material 3.

Supplementary Material 4.

Supplementary Material 5.

## Data Availability

The datasets generated and/or analyzed during the current study are not publicly available due to the inclusion of personal participant data that require anonymization, but are available after anonymization by the corresponding author on reasonable request.

## Abbreviations

PENG: PEricapsular Nerve Group block
METC: Medical Ethics Review Committee
CROSS: Checklist for Reporting of Survey Studies guidelines
StaRI: Standards for Reporting Implementation Studies guidelines
NvA: Nederlandse Vereniging voor Anesthesiologie (Dutch National Anesthesiology Society)
IQR: Interquartile ranges
AAPOR: American Association for Public Opinion Research
