# Supplementary material for: Attitudes of anesthesiologists towards implementation of PENG block in non-operative treatment of hip fractures in the Netherlands: a national survey study
Source: BMC Anesthesiol. 2026 May 29;26:451. doi: 10.1186/s12871-026-03957-y (PMC13410592; doi:10.1186/s12871-026-03957-y)
Supplement: Supplementary file 1 — Supplementary Material 1. [file 12871_2026_3957_MOESM1_ESM.docx]

### Supplementary files

**Supplementary file 1.** Study protocol as accepted by the Medical Ethics Review Committee (METC) of the Amsterdam UMC.

**(for applications for non-WMO declarations)**

Study protocol

**All information must match the information you enter in Research Manager**

**General information**

| **Title** | Attitudes of anesthesiologists towards implementation of PENG-block in non-operative treatment of hip fractures in the Netherlands: a national survey study. |
| --- | --- |
| **Date** | 11-02-2025 |
| **Version number** | Version 1.1 |
| **Submitter** | MI Lommerse, HC Willems, HJ Schuijt |
| **Coordinating researcher** | MI Lommerse, [m.i.lommerse@amsterdamumc.nl](mailto:m.i.lommerse@amsterdamumc.nl) |
| **Primary researcher(s)** | HC Willems, [h.c.willems@amsterdamumc.nl](mailto:h.c.willems@amsterdamumc.nl); HJ Schuijt, [h.schuijt@antoniusziekenhuis.nl](mailto:h.schuijt@antoniusziekenhuis.nl) |
| **Executing entity** | Amsterdam UMC |

**Study information**

| **Rationale** | Hip fractures are a significant health concern, particularly among the older patient population. The incidence of hip fractures is high at around 18.500 patients annually in the Netherlands and these injuries are associated with serious morbidity and mortality (1). Given the severe impact on health and the high costs associated with treatment and rehabilitation, hip fractures represent a substantial burden on healthcare systems (2).  For frail patients, especially those with multiple comorbidities and advanced age, surgical intervention may not always be the best option. Non-operative management (NOM) can be considered under certain circumstances (3). In these cases, the primary focus shifts towards improving the quality of life, particularly towards the end of life, with a significant emphasis on effective pain management (4). The goal is to alleviate pain and enhance comfort without subjecting patients to the risks associated with surgery (5).  One of the recent advancements in pain management for patients undergoing NOM of hip fractures is the pericapsular nerve group (PENG) block combined with phenol for neurolysis (6). The PENG block targets the sensory nerves around the hip joint, providing significant pain relief without affecting motor function. This preservation of motor function is crucial for maintaining the patient's comfort and ability to perform activities of daily living (ADL). This innovative approach offers a promising alternative for pain relief that reduces the reliance on opioids (7).  The use of the PENG block with phenol for neurolysis has already begun to be implemented by individual anesthesiologists, albeit in small numbers. Therefore, there is a growing need to establish a comprehensive implementation guideline and a standardized pathway to ensure consistency and high standards in the application of this technique. A standardized guideline would not only ensure homogeneity in the quality of PENG block administration but also provide clear recommendations on when it is appropriate to use this technique and when alternative approaches should be considered. |
| --- | --- |
| **Aim** | Given that the implementation of the PENG-block with phenol has already begun in a limited capacity across the Netherlands, it is crucial that any future implementation guidelines consider the current stage of this process. Notably, in current practice it is often the case that only one or two anesthesiologists within one hospital have administered the PENG-block, while their direct colleagues have refrained from its use. Therefore, the aim of this study is twofold: first, to explore and understand the expectations, experiences, and attitudes of anesthesiologists who are currently administering the PENG-block, and second, to compare these findings with the perspectives of anesthesiologists who have not yet adopted this practice. The study seeks to identify key technical and logistical factors that influence the acceptance and implementation of this pain management technique from the perspective of anesthesiologists. Hereby, the study aims to further current clinical implementation attempts and facilitate broader adoption of this technique in hospital settings. The following research question will be answered:  What barriers and facilitators do anesthesiologists-pain specialists identify in the implementation of the PENG-block regarding the use of the PENG-block with phenol for pain management in non-operative patients with proximal femur fractures?  Secondary questions include:   1. To what extend do anesthesiologists perceive the use of the PENG block with phenol as acceptable in the context of palliative care? 2. To what degree is successful implementation of the PENG block with phenol within existing palliative care practices perceived as feasible by anesthesiologists? 3. How appropriate do anesthesiologists perceive the PENG-block with phenol for managing pain in non-operatively managed hip fracture patients in palliative care? |
| **Study design** | This study employs a quantitative approach to a survey study design to provide a comprehensive understanding of anesthesiologists' experiences and perceptions regarding the use of the PENG-block with phenol in palliative care settings.  In order to comprehensively address the research questions, we have composed an implementation sciences-based model through which our research questions have been framed. *Figure 1* illustrates the combination of a determinant model as proposed by Geerligs et al. (8) with an evaluation model defined by Proctor et al. (9). In Geerligs et al.’s determinant model, the focus on the interaction between the system, staff, and intervention domains offers a framework for understanding the unique challenges and facilitators in the context of a hospital setting, making it the preferred model for this study. Additionally, Proctor et al.’s model is aimed at evaluating the implementation process through defining outcomes from three categories: implementation, service and client. This study adopts three of Proctor et al.’s implementation outcomes as thematic base of the survey questions:   - Acceptability: How well the PENG-block is received by anesthesiologists. - Feasibility: The practicality of implementing the PENG-block within the hospital setting. - Appropriateness: The perceived relevance and suitability of the PENG-block for managing pain in non-operative managed hip fracture patients.   These two models have both been used separately in a number of different implementational studies in a hospital setting, making their combination an appropriate conceptual foundation around which the survey questions will be framed.  Participants will provide informed consent prior to participation, with assurances of confidentiality and the right to withdraw from the study at any time without consequences. |
| **Study population** | Anesthesiologists-pain specialists in the Netherlands. |
| **Aimed number of participants in Amsterdam UMC** | ±15 |
| **Aimed number of participants in the Netherlands** | A total of 192 anesthesiologists-pain specialist are registered in the Netherlands. However, we expect survey participation rates of 60-70% resulting in a total of ±125 patients. |
| **Inclusion criteria** | Inclusion criteria for participants are anesthesiologists-pain specialists, regardless of their amount of experience with PENG-blocks, who are willing to consent to participate in the survey. |
| **Exclusion criteria** | Exclusion criteria are anesthesiologists-pain specialists without ability to participate in or complete the survey, or anesthesiologists (including residents and fellows) with a focus on other subspecialties. |
| **Number of participants/ sample size** | Based on a population of 192 anesthesiologists-pain specialists in the Netherlands, we expect survey participation rate of 65% which results in a total of 125 participants. |
| **Participant recruitment** | Recruitment of participants for this study will be conducted through a national PENG-consortium of which the researchers are a member. This national consortium has approximately 25 members throughout the Netherlands, all with an interest in furthering scientific and clinical impact of PENG and other non-operative palliative pain care management structures. A selection of consortium members will participate in the focus group and will be approached via email. Potential survey participants will also be recruited via email. This email will provide an introduction, online consent form for the survey and a link to the survey. After the survey has been distributed, a reminder mail will be sent after two weeks to those who have not started or completed the survey. |
| **Intervention** | The intervention in this study is an online survey that will be distributed across all anesthesiologists-pain specialists in the Netherlands. As stated above, survey questions will be categorized and divided according to the implementation outcomes acceptability, feasibility and appropriateness. While the majority of the survey questions are quantitative in nature (e.g. Likert-scale questions, close-ended questions, visual analogue scales and multiple-choice questions), at the end of each theme-specific survey item, a free-text question will be included. This final open-ended question invites participants to share any additional thoughts or topics they feel are relevant but were not addressed in the preceding survey questions.  Survey development will be performed by 3 researchers (ML, HW and HJS) and checked by 3 experts on relevance (RS and JWK). The survey will be pretested with 5 anesthesiologists-pain specialists to improve clarity.  The survey will be distributed online to all potentially eligible anesthesiologists. A distribution link will be sent by email to a survey in the digital survey system Research Survey. Potentially eligible participants will be sent a reminder 2 weeks after the initial distribution. |
| **Standard care** | Not applicable. |
| **Study endpoints** | Following the theoretical framework as illustrated in figure 1, the primary endpoint of our study consist of a number of barriers and facilitators categorized according to Geerligs et al.’s framework. Secondary endpoints include the implementation outcomes as described in the evaluation framework of Proctor et al. See T*able 1* for an explanation of how endpoints will be measurement. |

| **Implementation outcome** | **Definition Proctor et al.** | **Outcome measurement and data source** |
| --- | --- | --- |
| *Acceptability* | ‘The extent to which anesthesiologists perceive the use of the PENG block with phenol as satisfactory and agreeable in the context of palliative care.’ | Survey items assessing perceived ease of use, complexity of the technique and acceptability of the protocol, extend of administrative tasks, willingness to recommend its use, perceived benefits versus risks, patient friendliness, and acceptability on a five point Likert scale. |
| *Feasibility* | ‘The degree to which the PENG block with phenol can be successfully implemented within existing palliative care practices by anesthesiologists.’ | Survey items evaluating availability of resources, site readiness, technical skills required and availability of anesthesiologist expertise or training opportunities, fit into the overall care process, presence of established protocols and pain management strategies for non-operative management of hip fractures, the likelihood of integrating the technique into routine practice, previous experience with protocol implementation, most important perceived barriers and facilitators to influence feasibility and feasibility on a five point Likert scale. |
| *Appropriateness* | ‘The perceived relevance and suitability of the PENG block with phenol for managing pain in non-operatively managed hip fracture patients in palliative care.’ | Survey items addressing perceived quality of current evidence, patient-specific suitability, tension for change, openness to adopting new techniques and protocols, barriers and facilitator influencing appropriateness perceived as most important, appropriateness on five point Likert scale. |

**Table 1.** *Overview of implementation outcomes, its definitions and corresponding research questions, outcome measurements and data sources.*

| **Study parameters** | The following additional study parameters will be extracted:   - Demographics of the anesthesiologists-pain specialists: age, current position and role, type and size of institution; - Patient population: typical patient profile (age, gender, comorbidities) for those receiving PENG-blocks; - Professional background: frequency of dealing with palliative care patients, number of PENG-blocks performed, experience with other regional anesthesia techniques, additional training or certifications in pain management or palliative care. |
| --- | --- |
| **Statistical analysis** | The survey data will be analyzed using descriptive and inferential statistics, providing a quantifiable measure of anesthesiologists' experiences and perceptions. Survey responses will be analyzed using statistical software (SPSS, version 27; IBM Corporation) to identify trends and significant factors related to the implementation of the PENG-block. The open-ended questions that provide qualitative data will be investigated using a thematic analysis of the results. For this process, NVivo (version 12; QRS International) will be used. Triangulation will be performed using the Pillar Integration Process (PIP) technique as described by Johnson et al. (11). |
| **Burden on participants** | The burden for participants in this study involves completing the online survey, which will take approximately 15 minutes. |
| **Risk for participants** | The risks to participants are minimal. Participants are asked to share their experiences and opinions, which poses no physical risks. |
| **Benefits to participation in research** | Participation in the study provides anesthesiologists with the opportunity to contribute to the development of national guidelines for the implementation of the PENG block with phenol in palliative care settings. This can potentially improve the quality of care and pain management for non-operatively managed patients with hip fractures, benefiting the broader medical community and patient outcomes. |
| **Disadvantages to participation in research** | The main disadvantage for participants is the time commitment required to complete the survey. There are no other significant disadvantages associated with participation. |
| **Participant reimbursements** | Participants will not receive any reimbursements for participation in the study. |
| **Administrative aspects** | Based on a Data Management Plan (DMP), data and other records obtained will be handled in compliance with institutional regulations and ethical standards. To preserve participant confidentiality, data will be pseudonymized using a coding system and stored securely. Following the study's completion, the data will be kept for ten years before being safely destroyed. |
| **Publication policy and amendments** | The study's findings will be presented at relevant conferences and published in peer-reviewed medical journals. Before being put into action, any modifications to the study protocol will be sent to the METC for assessment and approval. |
| **Other points of interest for the Non-WMO Assessment Committee** |  |
| **References** | 1. Audit DHF. DHFA JAARRAPPORT. Dutch Institute for Clinical Auditing; 2022.  2. Cooper C, Campion G, Melton LJ, 3rd. Hip fractures in the elderly: a world-wide projection. Osteoporos Int. 1992;2(6):285-9.  3. Sing CW, Lin TC, Bartholomew S, Bell JS, Bennett C, Beyene K, et al. Global Epidemiology of Hip Fractures: Secular Trends in Incidence Rate, Post-Fracture Treatment, and All-Cause Mortality. J Bone Miner Res. 2023;38(8):1064-75.  4. Loggers SAI, Willems HC, Van Balen R, Gosens T, Polinder S, Ponsen KJ, et al. Evaluation of Quality of Life After Nonoperative or Operative Management of Proximal Femoral Fractures in Frail Institutionalized Patients: The FRAIL-HIP Study. JAMA Surg. 2022;157(5):424-34.  5. Loggers SAI, Van Balen R, Willems HC, Gosens T, Polinder S, Ponsen KJ, et al. The Quality of Dying in Frail Institutionalized Older Patients After Nonoperative and Operative Management of a Proximal Femoral Fracture: An In-Depth Analysis. American Journal of Hospice and Palliative Medicine®. 2024;41(6):583-91.  6. Kwun-Tung Ng T, Chan WS, Peng PWH, Sham P, Sasaki S, Tsui HF. Chemical Hip Denervation for Inoperable Hip Fracture. Anesth Analg. 2020;130(2):498-504.  7. Smits RJH, Tillmans, L. C. M., Moll, A. C., Vissers, K. C. P. & van der Wal, S. E. I. . Pericapsulaire zenuwblokkade na een heupfractuur. Ned Tijdschr Geneeskd. 2020;166:1–6.  8. Geerligs L, Rankin NM, Shepherd HL, Butow P. Hospital-based interventions: a systematic review of staff-reported barriers and facilitators to implementation processes. Implementation Science. 2018;13(1):1--17.  9. Proctor E, Silmere H, Raghavan R, Hovmand P, Aarons G, Bunger A, et al. Outcomes for Implementation Research: Conceptual Distinctions, Measurement Challenges, and Research Agenda. Administration and Policy in Mental Health and Mental Health Services Research. 2011;38(2):65--76.  10. Von Elm E, Altman DG, Egger M, Pocock SJ, Gøtzsche PC, Vandenbroucke JP, for the SI. The Strengthening the Reporting of Observational Studies in Epidemiology (STROBE) Statement: Guidelines for Reporting Observational Studies. Epidemiology. 2007;18(6).  11. Johnson R, Grove A, Clarke A. Pillar Integration Process: A Joint Display Technique to Integrate Data in Mixed Methods Research. Journal of Mixed Methods Research. 2017;13:155868981774310.  12. Damschroder LJ. Clarity out of chaos: Use of theory in implementation research. Psychiatry Res. 2020;283:112461. |

**
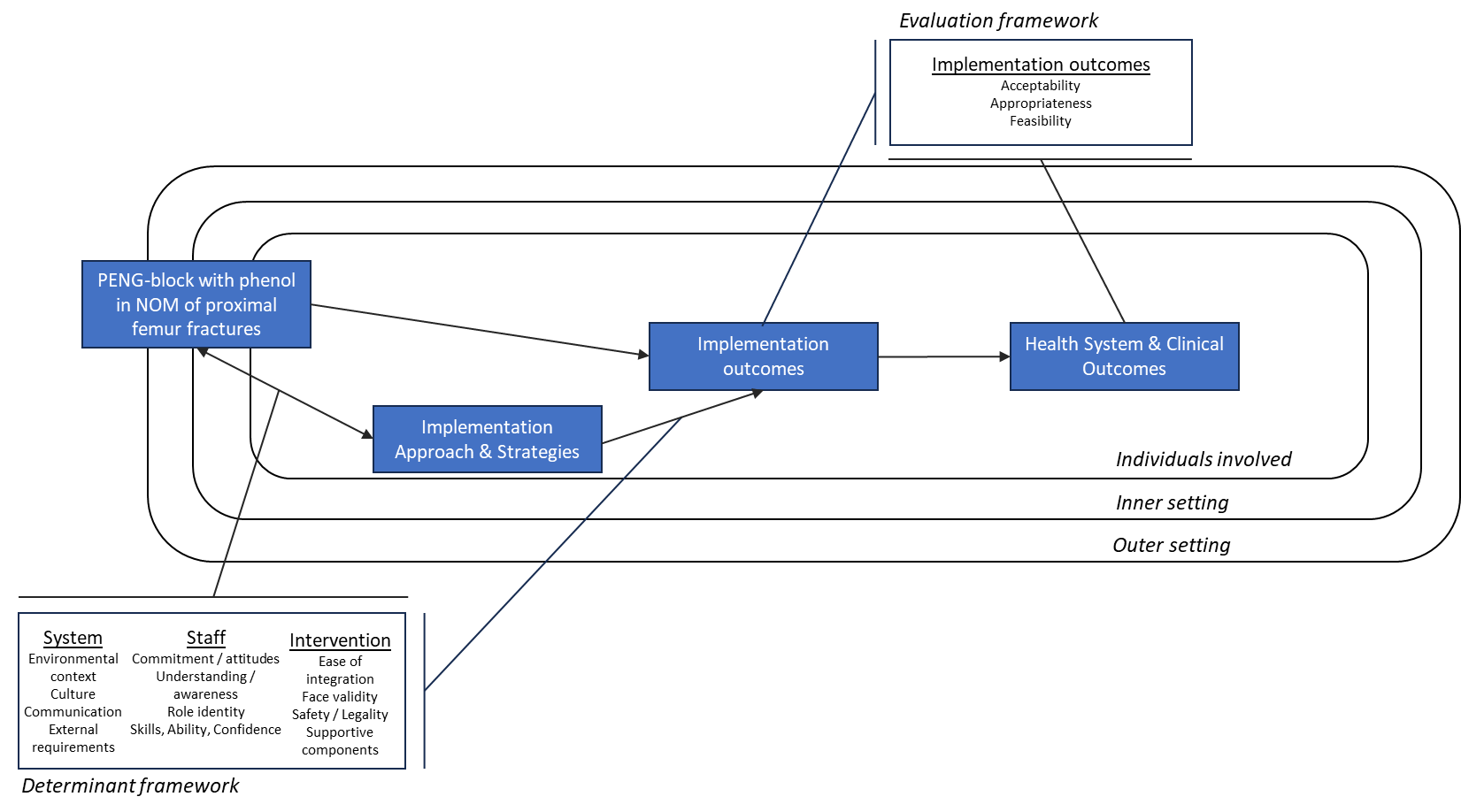
Figure 1.** *Combined model of determinant and evaluation frameworks by Geerligs et al. (8) and Proctor et al. (9). Taken from Damschröder (12).*
