# Supplementary material for: Attitudes of anesthesiologists towards implementation of PENG block in non-operative treatment of hip fractures in the Netherlands: a national survey study
Source: BMC Anesthesiol. 2026 May 29;26:451. doi: 10.1186/s12871-026-03957-y (PMC13410592; doi:10.1186/s12871-026-03957-y)
Supplement: Supplementary file 2 — Supplementary Material 2. [file 12871_2026_3957_MOESM2_ESM.docx]

**Supplementary file 2.** *Overview of implementation outcomes, including definitions, corresponding research questions, outcome measurements and data sources.*

| **Implementation outcome** | **Definition according to Proctor et al. [17]** | **Outcome measurement and data source** |
| --- | --- | --- |
| *Acceptability* | ‘The extent to which anesthesiologists perceive the use of the PENG block with phenol as satisfactory and agreeable in the context of palliative care.’ | Survey items assessing perceived ease of use, complexity of the technique and acceptability of the protocol, extend of administrative tasks, willingness to recommend its use, perceived benefits versus risks, patient friendliness, and acceptability on a five point Likert scale. |
| *Feasibility* | ‘The degree to which the PENG block with phenol can be successfully implemented within existing palliative care practices by anesthesiologists.’ | Survey items evaluating availability of resources, site readiness, technical skills required and availability of anesthesiologist expertise or training opportunities, fit into the overall care process, presence of established protocols and pain management strategies for non-operative management of hip fractures, the likelihood of integrating the technique into routine practice, previous experience with protocol implementation, most important perceived barriers and facilitators to influence feasibility and feasibility on a five point Likert scale. |
| *Appropriateness* | ‘The perceived relevance and suitability of the PENG block with phenol for managing pain in non-operatively managed hip fracture patients in palliative care.’ | Survey items addressing perceived quality of current evidence, patient-specific suitability, tension for change, openness to adopting new techniques and protocols, barriers and facilitator influencing appropriateness perceived as most important, appropriateness on five point Likert scale. |

[17] Proctor E, Silmere H, Raghavan R, Hovmand P, Aarons G, Bunger A, et al. Outcomes for Implementation Research: Conceptual Distinctions, Measurement Challenges, and Research Agenda. Administration and Policy in Mental Health and Mental Health Services Research. 2011;38(2):65--76.
