# Supplementary material for: Attitudes of anesthesiologists towards implementation of PENG block in non-operative treatment of hip fractures in the Netherlands: a national survey study
Source: BMC Anesthesiol. 2026 May 29;26:451. doi: 10.1186/s12871-026-03957-y (PMC13410592; doi:10.1186/s12871-026-03957-y)
Supplement: Supplementary file 3 — Supplementary Material 3. [file 12871_2026_3957_MOESM3_ESM.docx]

**Supplementary file 3a.** *Survey questions for participants with previous experience with PENG, coded according to corresponding implementation outcome(s).*

PENG-study: vragenlijst anesthesiologen

*Deze vragenlijst probeert te achterhalen welke barrières en faciliterende factoren er spelen rondom de implementatie van de Pericapsulaire Zenuwgroep (PENG)-blokkade* *met fenol in de non-operatieve setting bij patiënten met een heupfractuur.*

*Elke survey vraag is gecodeerd gebaseerd op het onderliggende thema waaraan deze vraag gekoppeld is. Legenda: Baseline gegevens*

*Haalbaarheid (“feasibility”)*

*Aanvaardbaarheid (“acceptability”)*

*Geschiktheid (“appropriateness”)*

1. **Baseline gegevens**
2. Wat is uw leeftijd?

(Leeftijd in jaren)

1. Wat is uw functie?

- Anesthesioloog-pijnspecialist
- Anesthesioloog (eventueel met ander subspecialisme)
- Anders, namelijk:

(Anders, namelijk)

1. Hoeveel jaren werkervaring heeft u?

(Geheel getal)

1. Heeft u zelf een of meerdere malen een PENG-blokkade uitgevoerd bij een patiënt met een niet-geopereerde heupfractuur?

- Ja
- Nee

*Hieronder cut-off van survey – expliciet voor anesthesisten* ***met*** *PENG-ervaring*

1. **Persoonlijke ervaring met PENG**
2. Hoeveel PENG-blokkades heeft u uitgevoerd voor patiënten met een niet-geopereerde **heupfractuur**?

- Minder dan 5
- 5-10
- 10-20
- Meer dan 20

1. Hoeveel PENG-blokkades heeft u uitgevoerd voor patiënten met een niet-geopereerde **mediale collumfractuur**?

- Minder dan 5
- 5-10
- 10-20
- Meer dan 20

1. Hoeveel PENG-blokkades heeft u uitgevoerd voor patiënten met een niet-geopereerde **pertrochantere fractuur**?

- Minder dan 5
- 5-10
- 10-20
- Meer dan 20

1. Welke hoeveelheid fenol gebruikt u voor de neurolyse?

- <7 ml
- 7-10 ml
- 11-15 ml
- Andere concentratie, namelijk:

(Andere concentratie, namelijk)

1. Welke techniek gebruikt u het meest bij deze patiënten?

- Middels 1 injectie (via PENG-techniek)
- Middels 2 injecties (via PENG-techniek + anterieure techniek)
- Andere, namelijk:

(Kunt u een reden geven voor uw keuze van techniek?)

(Anders, namelijk)

1. Hoe komt u aan uw kennis omtrent het PENG blok met fenol?

- Van collega(‘s)
- Wetenschappelijke publicatie
- Wetenschappelijke voordracht
- Instructie video (waaronder YouTube)
- Cursus
- Anders, namelijk:

(vrije tekst)

1. Heeft u behoefte aan extra of aanvullende training(en)? Zo ja, wat zou u specifiek willen verbeteren, uitbreiden of toevoegen?

- Nee
- Ja, namelijk:

(vrije tekst)

1. Na hoeveel keer het uitvoeren van een PENG-blokkade voelde u zich bekwaam over de techniek?

- Na 1-3 keer
- Na 4-6 keer
- Na 7-10 keer
- Na meer dan 10 keer

1. Wat was de reden voor uw gevoel van bekwaamheid of gebrek aan zekerheid over de techniek?

(vrije tekst)

1. Hoe zou u de algemene houding van uw vakgroep ten opzichte van de implementatie van PENG beschrijven?

- Zeer positief
- Positief
- Neutraal
- Negatief
- Zeer negatief

1. Wie zouden er volgens u een PENG-blokkade met fenol kunnen uitvoeren?

- Alleen anesthesioloog-pijnspecialist
- Alle anesthesiologen
- Naast anesthesiologen ook andere artsen, namelijk:

(Anders, namelijk)

1. **Stellingen over persoonlijke ervaring**
2. Ik vind de PENG-blokkade met fenol gemakkelijk toe te passen in mijn dagelijkse praktijk.

- Helemaal oneens
- Oneens
- Neutraal
- Eens
- Helemaal eens

1. De PENG-blokkade met fenol is een geschikte techniek voor het verlichten van pijn bij niet-operatieve patiënten met een **collumfractuur**.

- Helemaal mee oneens
- Mee oneens
- Neutraal
- Mee eens
- Helemaal mee eens

1. De PENG-blokkade met fenol is een geschikte techniek voor het verlichten van pijn bij niet-operatieve patiënten met een **pertrochantaire femurfractuur**.

- Helemaal mee oneens
- Mee oneens
- Neutraal
- Mee eens
- Helemaal mee eens

1. **Logistiek en systeem**
2. Waar worden de PENG-blokkades met fenol meestal geplaatst in uw ziekenhuis?

- Operatiekamer
- Pijnpolikliniek/behandelkamer
- Spoedeisende hulp
- Afdeling
- Recovery
- Andere locatie, namelijk:

(vrije tekst)

1. Hoeveel tijd zit er gemiddeld tussen de indicatiestelling en het plaatsen van een PENG-blokkade met fenol?

- Minder dan 12 uur
- 12-24 uur
- 24-36 uur
- Meer dan 36 uur

1. Mijn afdeling beschikt over voldoende middelen (inclusief scholing) om de PENG-blokkade met fenol effectief uit te voeren.

- Helemaal mee oneens
- Mee oneens
- Neutraal
- Mee eens
- Helemaal mee eens

1. Er is binnen de andere betrokken disciplines (bijv. geriatrie, traumatologie) voldoende kennis over de indicatie en verwachte resultaten voor het gebruik van de PENG-blokkade met fenol.

- Helemaal mee oneens
- Mee oneens
- Neutraal
- Mee eens
- Helemaal mee eens

1. Welke **barrières** beschouwt u als de grootste uitdaging bij het toepassen van de PENG-blokkade met fenol? (Meerdere antwoorden mogelijk)

- Gebrek aan bewijs
- Gebrek aan middelen
- Gebrek aan training en ervaring
- Logistieke uitdagingen (bijv. tijd, middelen)
- Administratieve lasten
- Onzekerheid over effectiviteit op lange termijn
- Andere, namelijk:

(Anders, namelijk)

1. Welke **positief beïnvloedende** **factoren** zouden volgens u het uitbreiden van de implementatie van PENG-blokkade met fenol vergemakkelijken? (Meerdere antwoorden mogelijk)

- Meer klinische studies en bewijzen
- Beschikbaarheid van middelen
- Opleidingsmogelijkheden
- Steun van collega's of ziekenhuismanagement
- Duidelijke richtlijnen en protocollen
- Andere, namelijk:

(Anders, namelijk)

1. **Afsluitend**
2. Heeft u ‘clinical pearls’ of tips die u zou willen delen met collega’s voor het veilig en succesvol uitvoeren van de PENG-blokkade?

(vrije tekst)

1. Heeft u nog andere opmerkingen of suggesties over het gebruik of de implementatie van PENG-blokkades met fenol bij de non-operatieve behandeling van patiënten met een proximale femurfractuur?

(Vrije tekst)

**Supplementary file 3b.** *Survey questions for participants without previous experience with PENG, coded according to corresponding implementation outcome(s).*

PENG-study: vragenlijst anesthesiologen

*Deze vragenlijst probeert te achterhalen welke barrières en faciliterende factoren er spelen rondom de implementatie van de Pericapsulaire Zenuwgroep (PENG)-blokkade* *met fenol in de non-operatieve setting bij patiënten met een heupfractuur.*

*Elke survey vraag is gecodeerd gebaseerd op het onderliggende thema waaraan deze vraag gekoppeld is. Legenda: Baseline gegevens*

*Haalbaarheid (“feasibility”)*

*Aanvaardbaarheid (“acceptability”)*

*Geschiktheid (“appropriateness”)*

1. **Baseline gegevens**
2. Wat is uw leeftijd?

(Leeftijd in jaren)

1. Wat is uw functie?

- Anesthesioloog-pijnspecialist
- Anesthesioloog (eventueel met ander subspecialisme)
- Anders, namelijk:

(Anders, namelijk)

1. Hoeveel jaren werkervaring heeft u?

(Geheel getal)

1. Heeft u zelf een of meerdere malen een PENG-blokkade uitgevoerd bij een patiënt met een niet-geopereerde heupfractuur?

- Ja
- Nee

*Hieronder cut-off van survey – expliciet voor anesthesisten* ***zonder*** *PENG-ervaring*

1. **Persoonlijke ervaring met PENG**
2. Hoeveel van uw collega’s binnen de vakgroep anesthesiologie kunnen een PENG-blokkade met fenol uitvoeren?

- Niemand
- Ongeveer een kwart van mijn collega’s
- Ongeveer de helft van mijn collega’s
- Ongeveer driekwart van mijn collega’s
- Alle collega’s

1. Hoe zou u de algemene houding van uw vakgroep ten opzichte van de implementatie van PENG beschrijven?

- Zeer positief
- Positief
- Neutraal
- Negatief
- Zeer negatief

1. Wie zouden er volgens u een PENG-blokkade met fenol kunnen uitvoeren?

- Alleen anesthesioloog-pijnspecialist
- Alle anesthesiologen
- Naast anesthesiologen ook andere artsen, namelijk:

(Anders, namelijk)

1. **Stellingen over persoonlijke ervaring**
2. De PENG-blokkade met fenol lijkt mij een complexe techniek.

- Helemaal oneens
- Oneens
- Neutraal
- Eens
- Helemaal eens

1. De PENG-blokkade met fenol lijkt mij een geschikte techniek voor het verlichten van pijn bij niet-operatieve patiënten met een proximale femurfractuur.

- Helemaal mee oneens
- Mee oneens
- Neutraal
- Mee eens
- Helemaal mee eens

1. Ik ben bereid de PENG-blokkade met fenol te integreren in de dagelijkse zorg voor palliatieve patiënten met proximale femurfracturen.

- Helemaal mee oneens
- Mee oneens
- Neutraal
- Mee eens
- Helemaal mee eens

1. **Logistiek en systeem**
2. Ik verwacht dat de PENG-blokkade met fenol eenvoudig kan worden geïntegreerd in de dagelijkse praktijk van mijn afdeling.

- Helemaal mee oneens
- Mee oneens
- Neutraal
- Mee eens
- Helemaal mee eens

1. Er is binnen de andere betrokken disciplines (bijv. geriatrie, traumatologie) voldoende kennis over de indicatie en verwachte resultaten voor het gebruik van de PENG-blokkade met fenol.

- Helemaal mee oneens
- Mee oneens
- Neutraal
- Mee eens
- Helemaal mee eens

1. Mijn zorginstelling is klaar voor de implementatie van de PENG-blokkade met fenol in de palliatieve zorg.

- Helemaal mee oneens
- Mee oneens
- Neutraal
- Mee eens

1. Welke **barrières** beschouwt u als de grootste uitdaging bij het toepassen van de PENG-blokkade met fenol? (Meerdere antwoorden mogelijk)

- Gebrek aan bewijs
- Gebrek aan middelen
- Gebrek aan training en ervaring
- Logistieke uitdagingen (bijv. tijd, middelen)
- Administratieve lasten
- Onzekerheid over effectiviteit op lange termijn
- Andere, namelijk:

(Anders, namelijk)

1. Welke **positief beïnvloedende** **factoren** zouden volgens u het starten met of uitbreiden van de implementatie van PENG-blokkade met fenol vergemakkelijken? (Meerdere antwoorden mogelijk)

- Meer klinische studies en bewijzen
- Beschikbaarheid van middelen
- Opleidingsmogelijkheden
- Steun van collega's of ziekenhuismanagement
- Duidelijke richtlijnen en protocollen
- Andere, namelijk:

(Anders, namelijk)

1. **Afsluitend**
2. Heeft u nog andere opmerkingen of suggesties over het gebruik of de implementatie van PENG-blokkades met fenol bij de non-operatieve behandeling van patiënten met een proximale femurfractuur?

(Vrije tekst)
